# Fingolimod in children with Rett syndrome: the FINGORETT study

**DOI:** 10.1186/s13023-020-01655-7

**Published:** 2021-01-06

**Authors:** Yvonne Naegelin, Jens Kuhle, Sabine Schädelin, Alexandre N. Datta, Stefano Magon, Michael Amann, Christian Barro, Gian Paolo Ramelli, Kate Heesom, Yves-Alain Barde, Peter Weber, Ludwig Kappos

**Affiliations:** 1 Neurologic Clinic and Policlinic, Departments of Medicine, Clinical Research, Biomedicine and Biomedical Engineering, University Hospital Basel and University of Basel, Petersgraben 4, 4031 Basel, Switzerland; 2 School of Biosciences, Cardiff University, Cardiff, CF10 3AX UK; 3 Clinical Trial Unit, Department of Clinical Research, University Hospital Basel, 4031 Basel, Switzerland; 4 Department of Pediatric Neurology and Developmental Medicine, University of Basel Children’s Hospital, 4056 Basel, Switzerland; 5 Medical Image Analysis Center (MIAC) AG, 4051 Basel, Switzerland; 6 Department of Biomedical Engineering, University of Basel, 4123 Allschwil, Switzerland; 7 Neuropediatric Unit, Pediatric Institute of Southern Switzerland, 6500 Bellinzona, Switzerland; 8 University of Bristol Proteomics Facility, Bristol, BS8 1TD UK

**Keywords:** Rett syndrome, Fingolimod, BDNF

## Abstract

**Background:**

Rett syndrome (RS) is a severe neurodevelopmental disorder for which there is no approved therapy. This study aimed to assess safety and efficacy of oral fingolimod in children with RS using a pre-post and case–control design.

**Methods:**

At the University of Basel Children’s Hospital, Basel, Switzerland, children with RS were included if they were older than 6 years and met the established diagnostic criteria of RS, including a positive MeCP2 mutation. Participants were observed 6 months before and after treatment and received 12 months of fingolimod treatment. Serum samples of 50 children without RS served as reference for brain-derived neurotrophic factor (BDNF) measurements. Primary outcome measures were safety and efficacy, the latter measured by change in levels of BDNF in serum/CSF (cerebrospinal fluid) and change in deep gray matter volumes measured by magnetic resonance imaging (MRI). Secondary outcome measure was efficacy measured by change in clinical scores [Vineland Adaptive Behaviour Scale (VABS), Rett Severity Scale (RSSS) and Hand Apraxia Scale (HAS)].

**Results:**

Six children with RS (all girls, mean and SD age 11.3 ± 3.1 years) were included. Serum samples of 50 children without RS (25 females, mean and SD age 13.5 ± 3.9 years) served as reference for BDNF measurements. No serious adverse events occurred. Primary and secondary outcome measures were not met. CSF BDNF levels were associated with all clinical scores: RSSS (estimate − 0.04, mult.effect 0.96, CI [0.94; 0.98], p = 0.03), HAS (estimate − 0.09, mult.effect 0.91, CI [0.89; 0.94], p <  0.01) and VABS (communication: estimate 0.03, mult.effect 1.03, CI [1.02; 1.04], p < 0.01/daily living: estimate 0.03, mult.effect 1.03, CI [1.02; 1.04], p < 0.01/social skills: estimate 0.07, mult.effect 1.08, CI [1.05; 1.11], p < 0.01/motoric skills: estimate 0.04, mult.effect 1.04, CI [1.03; 1.06], p = 0.02).

**Conclusions:**

In children with RS, treatment with fingolimod was safe. The study did not provide supportive evidence for an effect of fingolimod on clinical, laboratory, and imaging measures. CSF BDNF levels were associated with clinical scores, indicating a need to further evaluate its potential as a biomarker for RS. This finding should be further validated in independent patient groups.

**Trial Registration:**

Clinical Trials.gov NCT02061137, registered on August 27th 2013, https://clinicaltrials.gov/ct2/show/study/NCT02061137.

## Background

Rett syndrome (RS) is an X-linked neurodevelopmental disorder first described by Andreas Rett [1]. The classical variant is characterized by apparently normal development for the first 6–18 months, which shifts to early-onset stagnation (stage 1), followed by a period of regression (stage 2) of motor and language skills, stereotypic hand movements (91%), epilepsy (64.2%), autonomic dysfunction (episodic hyperventilation and breath-holding, and cardiorespiratory symptoms in up to 97%), and other symptoms, e.g., sleeping problems (80–94%), walking difficulties (79%), dysphagia (61%), scoliosis (56%), which is then followed by a plateau phase (stage 3) and a late motor deterioration phase (stage 4) [2–4]. The brains of patients with RS are smaller than those of healthy children but do not necessarily become progressively smaller over time [5]. There is a global reduction in both gray and white matter volumes [6]. Indeed, neuroimaging studies revealed a global reduction in cortical brain volumes, a reduction in volume of the basal ganglia, and a reduced global blood flow as common features of RS [7] and immunohistochemical studies indicated possible white matter abnormalities [8]. In 1999, mutations in the MeCP2 gene encoding X-linked methyl-CpG-binding protein 2 were identified as the cause of RS [9]. MeCP2 seems to be required to maintain neuronal function throughout life [10]. A key study that opened a window of opportunity for therapeutic interventions in RS showed a reversal of symptoms in a genetic mouse model after restoring MeCP2 [11].

Fingolimod is an oral drug that acts as a modulator of four of the five sphingosine-1 phosphate (S1P) receptors and is known to penetrate the blood–brain barrier [12]. Fingolimod was approved 10 years ago as a disease-modifying treatment for adults and, more recently, also for children with relapsing–remitting multiple sclerosis [13–16]. In mice lacking MeCP2, fingolimod (FTY720) treatment increased the levels of brain-derived neurotrophic factor (BDNF) and volume of deep and cortical gray matter and improved disease symptoms [17, 18]. BDNF is a member of the neurotrophin family; transcription is regulated by neural activity and its levels are known to be very low within the brain [19]. The significance of altered BDNF expression in RS has been demonstrated by providing evidence for a functional interaction between MeCP2 and BDNF [18].

This study aims to assess safety and efficacy of oral fingolimod in children with RS.

## Methods

### Participants

The study was performed at the Department of Pediatric Neurology and Developmental Medicine at the University of Basel Children’s Hospital, Basel, Switzerland. The study was designed as an open-label phase I/II clinical trial with a pre-post and case–control design. Participants served as their own controls: Each participant was followed up for 6 months before treatment (M0-6), was thereafter treated for 12 months (M6-18), and was additionally followed up for another 6 months (M18-24). The study visit overview is shown in Additional file 1. For this first exploratory proof-of-concept study, we planned to recruit 6 patients with RS. Children were included in the study (treatment group) if they fulfilled the diagnostic criteria for RS [20], were in stages II–IV [3, 21], had a confirmed MeCP2 mutation, were older than 6 years, and if parents had provided written informed consent. All study participants with RS were treated with fingolimod. Age and weight limitations as well as dosing considerations were based on pharmacokinetic modeling (performed by Novartis Pharma Schweiz AG): A dose of 0.5 mg was given for participants with a body weight over 40 kg, 0.25 mg for participants with a body weight of 15–40 kg.

To obtain reference values in a pediatric population, serum BDNF levels were measured in blood samples from neurologically healthy children (HC), aged 6–20 years, from whom blood had been drawn due to a routine surgical/gastrointestinal intervention.

### Serum and CSF sampling and analysis

Serum and CSF samples for biomarkers were obtained at baseline (BL), first dose visit (FD, M6), after 6 months of treatment (M12), and at the end of the treatment period (M18). CSF sampling was performed under a short general anesthesia with intravenous propofol.

Samples were analyzed using the Simoa BDNF Discovery Kit and a Simoa HD-1-Analyzer. All serum samples were run twice, all CSF samples three times. The mean of all results was taken as the final result for each time point.

Analysis of CSF using liquid chromatography-mass spectrometry (LCMS): We prioritized the possible identification of BDNF by digesting recombinant BDNF with trypsin and analyzing the resulting peptides by LCMS using an Orbitrap Fusion Mass Spectrometer (Thermo Scientific, detailed methods available on request). To increase the possibility of identifying BDNF in vivo, mass/charge (m/z), charge (z), and retention time (RT) values for peptides identified from the recombinant BDNF were then used to generate a priority inclusion list and the LCMS analysis was repeated using CSF samples as above, but the mass spectrometer was set to prioritize the fragmentation of peaks matching the inclusion list properties. Next, we analyzed CSF samples that had been spiked with increasing amounts of recombinant BDNF (ranging from 10 pg/ml to 18 ug/ml) using the inclusion list workflow described above to assess the threshold at which BDNF becomes detectable within CSF using our LCMS system.

We also measured neurofilament light chain (NfL) as a specific marker of neuroaxonal damage [22–24] in all serum and CSF samples, as described previously [22], using the aliquots obtained for the BDNF analysis.

For reasons of safety, blood was sampled (liver enzymes, white blood cells/lymphocyte count) at M0 (BL), M6 (FD), M7, M9, M12, M15, M18 (end of dose), and M24.

Levels of fingolimod (FTY-720 and FTY-720-P) in serum were measured before and six hours after administering the first dose, as well as one month after the first dose (M7) and after one year of treatment (M18) using mass spectrometry by WuXi AppTec (bioanalytical data report, property of Novartis Pharma Schweiz AG).

### Clinical and imaging assessments

In addition to the standard clinical assessments, clinical scores were assessed at M0, M6, M9, M12, M15, M18, M21, and M24 in patients and their families. These scores included the Vineland Adaptive Behaviour Scale (VABS) [25], the Rett Severity Scale (RSSS) [26], and the Hand Apraxia Scale (HAS) [27]. VABS addresses clinical skills in communication (67 questions), daily living (92 questions), socialization (66 questions), and motor functions (36 questions). Possible answers are 0 (no/never), 1 (sometimes), 2 (yes, regularly). Therefore, possible scores range from 0 to 522, a higher score reflecting a higher level of development. RSSS evaluates the clinical manifestation of RS with regards to onset, growth, motor skills, communication skills, and other symptoms (respiration, autonomic function, onset of stereotypies, epilepsy, and seizures). Possible scores range from 1 to 50, a higher score reflecting onset at earlier age and more impairment due to RS. HAS assesses 10 tasks (e.g., drinking from a cup) and the total score reflects the sum of all items (every task counts for 1 point) that a child is unable to do or does less than one-fourth of the time. Possible scores range from 0 to 10, a higher score reflecting a worse hand function.

Magnetic resonance imaging (MRI) was performed at M0, M6, M12, and M18 (with a short general anesthesia with intravenous propofol) on a 3.0 T magnetic resonance scanner (Magnetom Skyra, Siemens Healthineers, Germany). Imaging included an axial, coronal, and sagittal T1-weighted scout image to ensure correct head positioning followed by 3D T1w and 3D T2 SWI sequences. Deep gray matter volumes were segmented on the 3D T1w data (spatial resolution 1 × 1 × 1 mm^3^) measured using FSL-First (FSL version 5.0) as described previously [28, 29].

As an additional exploratory analysis, change in cerebral blood flow (cortex and deep gray matter structures (Basal Ganglia, Thalamus, Hippocampus) was measured by using pulsed arterial spin labeling technique (pASL) combined with a 2D EPI read-out (slice thickness 5 mm with 1.25-mm gap, in-plane resolution 3.0 × 3.0 mm^2^ interpolated to 1.5 × 1.5 mm^2^) [30, 31].

At M0, M6, and M18 a polysomnographic/electroencephalographic investigation was performed and was quantified using a modified grand total of EEG score [32] (see Additional file 2). The grand total of EEG score reflects the visual assessment of an EEG, taking into account frequency and reactivity of background activity, paroxysmal activity, focal disturbances, and sharp wave activities. The total score ranges from 1 to 31, a higher score reflecting a more abnormal finding (total score of 1 would be a normal EEG). The score was modified to meet the needs of children with RS: the major changes applied compared to the original score were the broader possible findings of paroxysmal activity and the replacement of sharp wave activity by sleep patterns. Breathing patterns were assessed clinically.

### Outcome measures

Primary outcome measures:

1. Efficacy: change in levels of BDNF in serum/CSF (cerebrospinal fluid) and change in deep gray matter volumes (thalamus, caudate, putamen, pallidum, hippocampus, amygdala and accumbens) measured by MRI.
2. Safety: white blood cell/lymphocyte counts, liver enzymes, and occurrence of any (serious) adverse events ((S)AE).

Secondary outcome measures:

1. Efficacy: Clinical scores (Vineland Adaptive Behaviour Scale, Rett Severity Scale and Hand Apraxia Scale), EEG patterns (grand total of EEG score), and breathing patterns.

Exploratory outcome measure:

1. Levels of NfL in serum/CSF as a biomarker for neuroaxonal loss.
2. Change in cerebral blood flow (cortex and deep gray matter structures (basal ganglia, thalamus, hippocampus)) measured by MRI.

### Statistical analysis

BDNF and NfL levels in serum were compared between patients with RS and healthy children (HC). Among the RS patients, we compared the average of the two measurements at M0 and M6 (untreated time period). In one patient there was no measurement at M6. In this case only the value at M0 was used. For BDNF p-values were estimated using a linear model, adjusted for thrombocytes (Tc) and hematocrit (Hct) at the same visit, as published previously [33]. BDNF was log-transformed prior to analysis. NfL was modeled in a robust model using the R-package robustbase (setting = “KS2014”). The p-values were adjusted for multiple comparisons using the Holm-Bonferroni method.

Pearson’s correlation was calculated for BDNF serum and CSF levels after averaging all measurements of each patient.

Changes between M0 and M6 (before treatment) and M6 and M18 (with treatment) in BDNF and NfL, serum and cerebrospinal fluid (CSF) levels, and for clinical scores (VABS, RSSS, HAS) were assessed with paired t-tests. Only those RS patients were evaluated for whom data from measurements at M0, M6, and M18 were available (serum N = 5; CSF N = 4).

The association between BDNF and NfL in serum and CSF and the outcome scores at visits M0, M6, M12, and M18 were assessed in a simple linear model using generalized estimating equations (GEE).

Brain volume changes between BL and M6 (before treatment) and M6 and M18 (treatment with fingolimod) were assessed. The average change between BL and M6 as well as between M6 and M18 was estimated together with the 95% confidence interval and assessed in a paired t-test.

For the concurrent association between BDNF/NfL and regional brain volumes at BL, M6, M12, and M18 and between cortical and deep gray matter perfusion, brain volumes and perfusion served as dependent, BDNF/NfL as independent variables. The estimates were adjusted for patient age. The association was assessed in a linear model using GEE.

### Ethical approval

The study was approved by the local ethics committee (EKNZ, Ref. 197/12) and by the Swiss agency for therapeutic products Swissmedic (Ref. 2013DR1109). Written informed consent was obtained from all parents of participating children [RS (all) and HC (if children were younger than 14)] and from all healthy children (age 6–10 oral informed consent only, but written informed consent from parents).

## Results

Six children with RS (all female, mean and SD age 11.3 ± 3.1 years) and 50 HC (25 females, mean and SD age 13.5 ± 3.9 years) were included and completed the study according to the protocol. Baseline characteristics of children with RS are shown in Table 1, those of HC in Additional file 3. No serious adverse events occurred. All participants were compliant with regards to the study medication even if oral intake was hindered by dysphagia (see Table 1).

**Table 1:** Patients characteristics at baseline (BL)

	All	Individual patients					
Number of patients	6	001	002	003	004	005	006
Number of females (%)	6 (100)						
Age (mean and SD) in years	11.3 (3.1)	12	13	12	10	6	15
Disease duration (mean and SD) in years	10.4 (2.5)	10	12	10	9	5	12
Height (mean and SD) in cm	143.8 (12.8)	152	123.5	144.4	147	122	155
Weight (mean and SD) in kg	50.5 (24.8)	43.6	28.5	93.40	48	27	56
BMI (mean and SD)	23.9 (10.5)	19.0	15.7	44.7	22.2	18.1	23.3
Fingolimod dose (mg)		0.5	0.25	0.5	0.5	0.25	0.5
Fingolimod administration		Normal (sometimes capsule broken with teeth)	PEG (opened capsule and given with liquid)	Normal	Normal	Opened capsule and given with liquid or meal	Opened capsule and given with liquid or meal
Mutation MeCP2 confirmed (%)	6 (100)	+	+	+	+	+	+
Rett Stage (Hagberg)		III	III	III	IV	III	II
Seizures (%)	4 (66.7)	+	+	−	+	+	−
AED	5 (83.3)	+	+	+ (mood stabili − ser)	+	+	−
Hyper-hypo-ventilation (%)	4 (66.7)	+	+	+	−	+	−
*Main symptoms at BL:*							
hand skills loss (%)	6 (100.0)	+	+	+	+	+	+
spoken language loss (%)	6 (100.0)	+	+	+	+	+	+
gait abnormalities (%)	6 (100.0)	+	+	+	+	+	+
stereotypical moves (%)	5 (83.3)	+	+	–	+	+	+
*Vineland Adaptive Behaviour Scale*		41	44	89	47	45	160
*Rett Symptom Severity Scale*		18	25	6	18	28	4
*Hand Apraxia Scale*		7	10	9	10	10	3
MRI-Inspection		Normal	Mode-rate generalized atrophy	Micro-cephaly	Normal	Decent generalized atrophy	Normal
BDNF serum in ng/ml							
		16.79	26.59	23.95	20.77	38.16	25.90
Mean and SD	25.4 (7.2)						
Median [IQR]	24.9 [21.6, 26.4]						
BDNF csf in pg/ml							
		0.265	0.318	7.309	0.266	2.581	1.783
Mean and SD	2.1 (2.7)						
Median [IQR]	1.1 [0.3, 2.4]						
NfL serum in pg/ml							
		10.5	17.3	6.3	8.6	5.0	12.1
Mean and SD	10.0 (4.4)						
Median [IQR]	9.6 [6.9, 11.7]						
NfL CSF in pg/ml							
		305.3	1003.4	201.3	292.1	237.8	302.3
Mean and SD	390.4 (303.1)						
Median [IQR]	297.2 [251.4, 304.6]						

*SD* standard deviation, *AED* antiepileptic drug

At BL, serum BDNF levels in children with RS did not differ from those in HC [p = 0.708 (all HC, N = 50)/p = 1 (only HC girls with age<15, N = 14)], nor did serum NfL levels at BL [p = 1 (all HC, N = 50)/p = 1 (only HC girls aged <15, N = 14)].

Serum BDNF levels at BL (ng/ml mean and SD) in patients with RS were 25.4 ± 7.2, in HC 22.14 (5.92). Serum NfL levels at BL (pg/ml mean and SD) in RS were 10.0 ± 4.4, in HC 9.95 ± 5.56. CSF BDNF level at BL (pg/ml mean and SD) in RS was 2.1 ± 2.7, CSF NfL level at BL (pg/ml mean and SD) in RS was 390.4 ± 303.1. There was no clear evidence of an age or gender effect regarding BDNF and NfL in serum in 50 HC (Additional file 4). There was no evidence for a correlation between BDNF levels in serum and CSF (rho = 0.15, CI = [− 0.75; 0.86], p = 0.774).

There was no statistical evidence for a change in serum and CSF levels for BDNF and NfL over time (see Additional file 5). The average change within the clinical scales (VABS, RSSS, HAS) did not differ when compared before and during treatment (see Additional file 6).

No association was found between BDNF/NfL in serum and the clinical scores. CSF BDNF was associated with all clinical scores (Table 2): Increased RSSS and HAS (for both scales higher scores are less favorable) were associated with lower BDNF in CSF, (RSSS: estimate − 0.04, mult.effect 0.96, CI [0.94; 0.98], p = 0.03/HAS: estimate − 0.09, mult.effect 0.91, CI [0.89; 0.94], p<0.01) whereas higher VABS (higher scores are more favorable) are associated with higher BDNF (VABS communication: estimate 0.03, mult.effect 1.03, CI [1.02; 1.04], p<0.01/VABS daily living: estimate 0.03, mult.effect 1.03, CI [1.02; 1.04], p<0.01/VABS social skills: estimate 0.07, mult.effect 1.08, CI [1.05; 1.11], p<0.01/VABS motoric skills: estimate 0.04, mult.effect 1.04, CI [1.03; 1.06], p = 0.02).

**Table 2:** Association of BDNF and NfL in CSF and clinical scales

Clinical Score	estimate	Mult.effect	CI	p
BDNF				
RSSS	− 0.04	0.96	[0.94; 0.98]	0.03
HAS	− 0.09	0.91	[0.89; 0.94]	< 0.01
VABS communication skills	0.03	1.03	[1.02; 1.04]	< 0.01
VABS daily living skills	0.03	1.03	[1.02; 1.04]	< 0.01
VABS social skills	0.07	1.08	[1.05; 1.11]	< 0.01
VABS motor skills	0.04	1.04	[1.03; 1.06]	0.02
NfL				
RSSS	0.02	1.03	[1.00; 1.05]	0.28
HAS	0.03	1.03	[0.99; 1.08]	0.45
VABS communication skills	0.00	1.00	[1.00; 1.01]	0.8
VABS daily living skills	− 0.00	1.00	[0.98; 1.01]	0.75
VABS social skills	− 0.01	0.99	[0.97; 1.01]	0.53
VABS motor skills	− 0.01	0.99	[0.97; 1.00]	0.43

*RSSS* Rett Severity Scale, *HAS* Hand Apraxia Scale, *VABS* Vineland Adaptive Behaviour Scale

No association was found between NfL in CSF and the clinical scores (Table 2); nor were any consistent changes found by assessing the average change in regional brain volumes before and during treatment (see Additional file 7). BDNF in serum was associated with the volumes of nucleus accumbens (estimate − 20.76, SE 4.89, CI [− 30.355; 11.171], p<0.01) and hippocampus (estimate − 91.05, SE 43.22, CI [− 175.764;  − 6.333], p = 0.035). There was a strong association between increased NfL and a smaller deep gray matter volume (Table 3).

**Table 3:** Association between BDNF/NfL and deep gray matter volumes at the same visit, adjusted for age

Brain region	Source	Estimate	SE	CI	p
BDNF					
Thalamus	Serum	− 145.97	117.07	[− 375.429; 83.493]	0.212
	CSF	214.06	139.28	[− 58.925; 487.047]	0.124
Caudate	Serum	− 53.78	43.26	[− 138.569; 30.999]	0.214
	CSF	36.72	55.75	[− 72.561; 145.994]	0.510
Putamen	Serum	− 51.60	82.04	[− 212.397; 109.191]	0.529
	CSF	91.56	107.25	[− 118.650; 301.763]	0.393
Pallidum	Serum	− 24.99	30.76	[− 85.287; 35.303]	0.417
	CSF	59.34	39.40	[− 17.888; 136.572]	0.132
Hippocampus	Serum	− 91.05	43.22	[− 175.764; − 6.333]	0.035
	CSF	− 13.73	64.68	[− 140.512; 113.046]	0.832
Amygdala	Serum	− 16.06	23.46	[− 62.032; 29.920]	0.494
	CSF	16.23	27.50	[− 37.673; 70.130]	0.555
Accumbens	Serum	− 20.76	4.89	[− 30.355; − 11.171]	< 0.01
	CSF	15.93	11.83	[− 7.255; 39.124]	0.178
NfL					
Thalamus	Serum	− 79.96	34.05	[− 146.704; − 13.216]	0.019
	CSF	− 2.41	0.81	[− 3.994; − 0.829]	< 0.01
Caudate	Serum	− 37.81	12.57	[− 62.440; − 13.185]	< 0.01
	CSF	− 1.14	0.30	[− 1.735; − 0.554]	< 0.01
Putamen	Serum	− 33.13	24.04	[− 80.253; 13.985]	0.168
	CSF	− 1.45	0.44	[− 2.318; − 0.588]	< 0.01
Pallidum	Serum	− 24.58	8.60	[− 41.442; − 7.717]	< 0.01
	CSF	− 0.74	0.20	[− 1.127; − 0.349]	< 0.01
Hippocampus	Serum	− 56.13	14.14	[− 83.846; − 28.409]	< 0.01
	CSF	− 1.12	0.55	[− 2.195; − 0.048]	0.041
Amygdala	Serum	− 8.60	6.66	[− 21.652; 4.457]	0.197
	CSF	− 0.40	0.14	[− 0.682; − 0.124]	< 0.01
Accumbens	Serum	− 6.88	2.51	[− 11.803; − 1.948]	< 0.01
	CSF	− 0.11	0.10	[− 0.300; 0.081]	0.261

No association between BDNF (serum and CSF) and perfusion of cortex and hippocampus could be shown. We did observe an association between CSF BDNF (not for serum BDNF) and the perfusion of basal ganglia and thalamus (basal ganglia (estimate 0.94, SE 0.38, CI [0.199; 1.673], p = 0.013)/thalamus (estimate 3.11, SE 1.14, CI [0.867; 5.351], p < 0.01). There was also an association between increased NfL (pg/ml in serum and CSF) and a lower perfusion in all areas measured (Table 4).

**Table 4:** Association between BDNF/NfL and cerebral blood flow at the same visit, adjusted for age

Brain region	Source	Estimate	SE	CI	p
BDNF					
Cortex*	Serum	0.10	0.55	[− 0.984; 1.185]	0.856
	CSF	1.42	0.94	[− 0.415; 3.261]	0.129
Insula	Serum	0.35	0.59	[− 0.800; 1.505]	0.549
	CSF	1.19	1.00	[− 0.761; 3.145]	0.232
Occipital	Serum	− 0.20	0.74	[− 1.652; 1.260]	0.792
	CSF	2.27	1.44	[− 0.547; 5.091]	0.114
Parietal	Serum	0.06	0.49	[− 0.904; 1.033]	0.896
	CSF	1.59	0.87	[− 0.119; 3.309]	0.068
Temporal	Serum	− 0.25	0.55	[− 1.327; 0.823]	0.646
	CSF	1.21	1.02	[− 0.791; 3.219]	0.235
Basal ganglia	Serum	− 0.08	0.26	[− 0.589; 0.432]	0.763
	CSF	0.94	0.38	[0.199; 1.673]	0.013
Thalamus	Serum	0.02	0.77	[− 1.487; 1.535]	0.975
	CSF	3.11	1.14	[0.867; 5.351]	< 0.01
Hippo-campus	Serum	− 0.02	0.47	[− 0.950; 0.912]	0.967
	CSF	1.12	0.69	[− 0.224; 2.464]	0.102
NfL					
Cortex*	Serum	− 0.58	0.11	[− 0.796; − 0.368]	< 0.01
	CSF	− 0.01	0.01	[− 0.023; − 0.002]	0.018
Insula	Serum	− 0.40	0.11	[− 0.610; − 0.196]	< 0.01
	CSF	− 0.01	0.00	[− 0.017; − 0.005]	< 0.01
Occipital	Serum	− 0.91	0.14	[− 1.185; − 0.637]	< 0.01
	CSF	− 0.02	0.01	[− 0.034; 0.000]	0.049
Parietal	Serum	− 0.70	0.14	[− 0.970; − 0.434]	< 0.01
	CSF	− 0.10	0.01	[− 0.025; − 0.003]	0.011
Temporal	Serum	− 0.65	0.11	[− 0.861; − 0.449]	< 0.01
	CSF	− 0.01	0.01	[− 0.025; − 0.004]	< 0.01
Basal ganglia	Serum	− 0.38	0.07	[− 0.519; − 0.237]	< 0.01
	CSF	− 0.01	0.00	[− 0.014; − 0.003]	< 0.01
Thalamus	Serum	− 0.53	0.14	[− 0.810; − 0.251]	< 0.01
	CSF	− 0.01	0.01	[− 0.026; − 0.002]	0.019
Hippo-campus	Serum	− 0.47	0.10	[− 0.661; − 0.269]	< 0.01
	CSF	− 0.02	0.00	[− 0.023; − 0.008]	< 0.01

*Cortex: includes cortex of Insula, occipital, parietal, and temporal lobes. Frontal cortex evaluation was not possible due to susceptibility artefacts

CSF BDNF levels could not be confirmed by liquid chromatography-mass spectrometry.

The average change in the grand total EEG scores did not differ when comparing the periods before and during treatment. There was no change in breathing pattern abnormalities over the study period (polysomnography).

No SAEs occurred during the study period. There were 7 AE during the study period possibly related to the treatment (4/6 children, aphthosis (2), pharyngitis (1), viral gastroenteritis (1), and upper airway infections (3)). There was a slight increase in liver enzymes (see Additional file 8) and an expected decrease in white blood cells and in lymphocyte counts during the treatment phase (see Additional file 9).

Levels of fingolimod during the study phase were within the expected range, known from other fingolimod trials (confidential data from Novartis, not shown). For levels of FTY720/FTY720P, see Additional file 10.

## Discussion

This study details an attempt to use knowledge from a genetically defined animal model of Rett Syndrome and to apply it to the human condition. Mice lacking MeCP2 presented decreased levels of BDNF in brain areas critical for motor function, including the striatum and the cerebellum, and fingolimod has been shown to restore these levels [17, 18]. In addition, fingolimod improved motor coordination of MeCP2 null animals and increased the volume of the striatum [17], a brain area known to be reduced in animals lacking BDNF [34, 35]. In our study, fingolimod was well tolerated and safe over a treatment period of 12 months. This is in accordance with results of a phase III study in pediatric MS where fingolimod was also well tolerated [15]. Compliance with study medication was supported by the clear drop in the lymphocyte count and the FTY720/FTY720P levels measured in serum.

Serum BDNF levels at baseline did not differ between children with RS and HC.

We did not observe any consistent effects of the 12-month fingolimod treatment on serum or CSF BDNF levels or on deep gray matter volumes. Neither did we find any indication of a consistent effect on clinical, neurophysiological, and patient-reported outcomes. Thus, we were not able to reproduce the effects observed in the experimental model in the human disease.

When assessing the association between the levels of BDNF and deep gray matter volumes at all visits (using GEE), BDNF in serum, but not in the CSF, was associated with the volumes of the nucleus accumbens and hippocampus. The reason for this correlation is unclear given that the bulk of serum BDNF originates from platelets and ultimately megakaryocytes [36]. Interestingly, BDNF levels in CSF did correlate with all outcome scores, with better scores being associated with higher BDNF levels. These results suggest that, unlike the levels of BDNF in serum, those in CSF may represent a valuable biomarker for RS. However, this assumption is not only based on a very small patient population, but the levels of BDNF in CSF are also so low that the presence of BDNF could not be confirmed independently by LCMS.

RS is understood to be a neurodevelopmental, not a neurodegenerative disease [5]. This notion is supported by NfL levels in serum, a marker of ongoing or recent neuroaxonal damage [24]: NfL levels at BL did not differ between patients with RS and 50 HC and there was no relevant change in levels over time in the fingolimod-treated cohort. However, a clear association was noted between increased NfL and reduced volumes of nearly all brain areas, which is in line with previous findings in another patient population [23]. In addition, there was a strong association between increased NfL concentrations in serum and CSF and a lower perfusion in all measured areas, an observation possibly linked to the decreased, lower brain volumes.

Our study has several limitations. First, the number of RS patients was very small and the study is probably underpowered with regards to group comparisons of BDNF measurements by ELISA [33]. Secondly, our study design was not placebo controlled. Thirdly, given the lack of data on the use of fingolimod in young children, 6 years was set as the lower age limit for study inclusion. Lastly, while potentially important, the results related to measurements of BDNF levels in CSF must be interpreted with caution as they could not be independently validated with another method. The antibody-based technique used here has been pushed to the limit and, although the antibody used has been previously validated, the levels measured were below the detection threshold of LCMS.

## Conclusions

Fingolimod treatment in this patient group was safe. This study failed to support an effect of fingolimod on BDNF levels (serum and CSF) and on clinical/imaging outcome measures, however. The low levels of BDNF detected by ELISA in CSF correlated with all clinical outcome scores, thus indicating a need to further evaluate the possible role of BDNF in the pathogenesis of RS and its utility as a potential, novel biomarker for RS. This finding needs to be confirmed in independent patient samples and also subjected to rigorous biochemical validation.

## Supplementary Information

**Additional file 1.** Overview of visits during the study period. V = Visit; SC = Screening; BL = Baseline; FD = Fist Dose; M = Month; X = BDNF sapling and all endpoint variable measurements.

**Additional file 2.** Modified Grand Total of EEG Score (GTE-Score).

**Additional file 3.** Baseline characteristics of healthy control children.

**Additional file 4.** BDNF(A) and NfL (B) in HC plotted by age and gender. Data of patients aged >15 years are shaded.

**Additional file 5.** Change of BDNF and NfL before and under treatment.

**Additional file 6.** Changes of clinical scores over time. Change is indicated by absolute numbers of scores, eg. BL 7 and change over time -1, 3, -6: score changed from 7 to 6,9 and 3 respectively within the given time period. Changes over time in all scales and patients were not different in the intervals before during and after treatment. VABS = Vineland Adaptive Behaviour Scale; RSSS = Rett Severity Scale; HAS = Hand Apraxia Scale; M = Month of Study.

**Additional file 7.** Brain volume change before and under treatment.

**Additional file 8.** Liver enzymes over the study period. A: ASAT, B: ALAT, C: GGT.

**Additional file 9.** White blood cell count (leukocytes, A) and lymphocytes (B) over the study period.

**Additional file 10.** Concentration of FTY720P (A) and FTY720 (B) over the study period.

## Data Availability

The data are available from the corresponding author on reasonable request.

## Abbreviations

BDNF: brain-derived neurotrophic factor
CSF: cerebrospinal fluid
GEE: generalized estimating equations
HAS: Hand Apraxia Scale
HC: healthy children
LCMS: liquid chromatography–mass spectrometry
MRI: magnetic resonance imaging
NFL: neurofilament light chain
RS: Rett syndrome
RSSS: Rett Severity Scale
VABS: Vineland Adaptive Behaviour Scale
