# Supplementary material for: Fingolimod in children with Rett syndrome: the FINGORETT study
Source: Orphanet J Rare Dis. 2021 Jan 6;16:19. doi: 10.1186/s13023-020-01655-7 (PMC7789265; doi:10.1186/s13023-020-01655-7)
Supplement: Supplementary file 1 — Additional file 1. Overview of visits during the study period. V = Visit; SC = Screening; BL = Baseline; FD = Fist Dose; M = Month; X = BDNF sapling and all endpoint variable measurements. [file 13023_2020_1655_MOESM1_ESM.docx]

Additional file 1

| Before treatment (6 months) | | During treatment (12 months) | | | | | | After treatment (6 months) | |
| --- | --- | --- | --- | --- | --- | --- | --- | --- | --- |
| SC | BL | FD | treatment | | | | | extension | |
|  | M0 | M6 | M7 | M9 | M12 | M15 | M18 | M21 | M24 |
| V1 | V2 | V3 | V4 | V5 | V6 | V7 | V8 | V9 | V10 |
|  | X | X |  |  | X |  | X |  |  |

*Additional file 1: Overview of visits during the study period*

V=Visit; SC=Screening; BL=Baseline; FD=First Dose; M=Month; X BDNF sampling and all endpoint variable measurements
