# Supplementary material for: Fingolimod in children with Rett syndrome: the FINGORETT study
Source: Orphanet J Rare Dis. 2021 Jan 6;16:19. doi: 10.1186/s13023-020-01655-7 (PMC7789265; doi:10.1186/s13023-020-01655-7)
Supplement: Supplementary file 2 — Additional file 2. Modified Grand Total of EEG Score (GTE-Score). [file 13023_2020_1655_MOESM2_ESM.docx]

Additional file 2

| **1. Frequency of rhythmic background activity (during wakefulness)** |
| --- |
| 🔾 0 = > 9.0 Hz Please indicate:  🔾 1 = 8-9 Hz 🔾 Normal (age related)  🔾 2 = 7-8 Hz 🔾 Minimal Slowing (1-2 Hz less than normal)  🔾 3 = 6-7 Hz 🔾 Moderate Slowing (3-4 Hz less than normal)  🔾 4 = 4-6 Hz 🔾 Marked Slowing (>4 Hz less than normal)  🔾 5 = 1-4 Hz  🔾 6 = none |
| **2. Diffuse slow activity** |
| 🔾 0 = none  🔾 1 = intermittent theta  🔾 2 = intermittent theta + sporadic delta  🔾 3 = intermittent theta + intermittent delta  🔾 4 = continuous theta + delta  🔾 5 = continuous delta |
| **3. Reactivity of rhythmic background activity** |
| 🔾 0 = normal  🔾 1 = decreased on eye opening  🔾 2 = absent on eye opening  🔾 3 = absence of any reactivity |
| **4. Paroxysmal activity (during wakefulness and during sleep)** |
| 🔾 0 = none  🔾 3 = paroxysmal slow activity  Specify:………………………………………………………………  🔾 5 = paroxysmal epileptic activity  Specify:………………………………………………………………  🔾 Fokal spike or sharp waves  🔾 Generalized spike-wave or polyspike-wave  🔾 Multifocal spike and/or sharp-wave discharges  🔾 Continuus generalized (slow) spike-wave activity |
| **5. Focal abnormalities (during wakefulness) if abnormal:** |
| 🔾 0 = no focal abnormalities 🔾 right 🔾 left  🔾 1 = slight unilateral abnormalities 🔾 frontal  🔾 2 = slight bilateral abnormalities 🔾 temporal  🔾 3 = severe unilateral and slight contralateral 🔾 central  🔾 4 = severe bilateral 🔾 parietal  🔾 5 = multifocal 🔾 occipital |
| **6. Sleep** |
| 🔾 0 = Normal, well-defined vertex transients and sleep spindles  🔾 1 = Loss of sleep spindles and/or vertex transients  🔾 2 = Absent NREM sleep characteristics  🔾 3 = Absence of any sleep characteristics |
| **GTE-Score= sum+1.** |

*Additional file 2: Modified Grand Total of EEG Score (GTE-Score)*
