# Supplementary material for: Fingolimod in children with Rett syndrome: the FINGORETT study
Source: Orphanet J Rare Dis. 2021 Jan 6;16:19. doi: 10.1186/s13023-020-01655-7 (PMC7789265; doi:10.1186/s13023-020-01655-7)
Supplement: Supplementary file 3 — Additional file 3. Baseline characteristics of healthy control children. [file 13023_2020_1655_MOESM3_ESM.docx]

Additional file 3

|  | **Age 6-15** | **Age >15** | **Overall** |
| --- | --- | --- | --- |
| Number | 28 | 22 | 50 |
| Number of females (%) | 14 (50) | 11 (50) | 25 (50) |
| Number of males (%) | 14 (50) | 11 (50) | 25 (50) |
| Age (mean and sd) in years | 10.7 (2.8) | 17 (1.4) | 13.5 (3.9) |
| Regular visit |  |  | 50 (100) |
| Main reason for visit  - orthopedic surgery  - general surgery  - gastrointestinal intervention  -oto-rhino-laryngological intervention | 19 (67.9)  7 (25)  2 (7.1)  0 | 16 (72.7)  2 (9.1)  3 (13.6)  1 (4.5) | 35 (70)  9 (18)  5 (10)  1 (2) |
| serum BDNF (mean and sd) in ng/ml | 22.6 (5.6) | 21.5 (6.4) | 22.1 (5.9) |
| serum NfL (mean and sd) in pg/ml | 10.0 (5.9) | 9.9 (5.2) | 10.0 (5.6) |

*Additional file 3:*  Baseline characteristics of healthy control children
