# Supplementary figures and images for: Fingolimod in children with Rett syndrome: the FINGORETT study
Source: Orphanet J Rare Dis. 2021 Jan 6;16:19. doi: 10.1186/s13023-020-01655-7 (PMC7789265; doi:10.1186/s13023-020-01655-7)

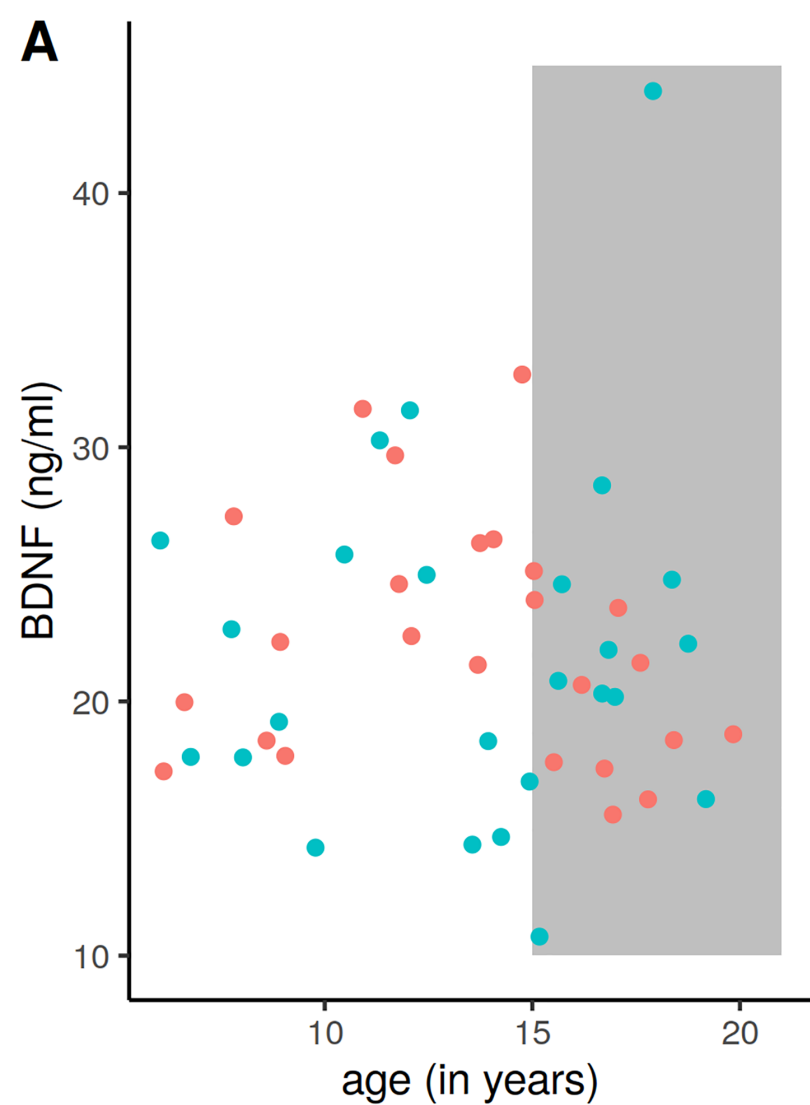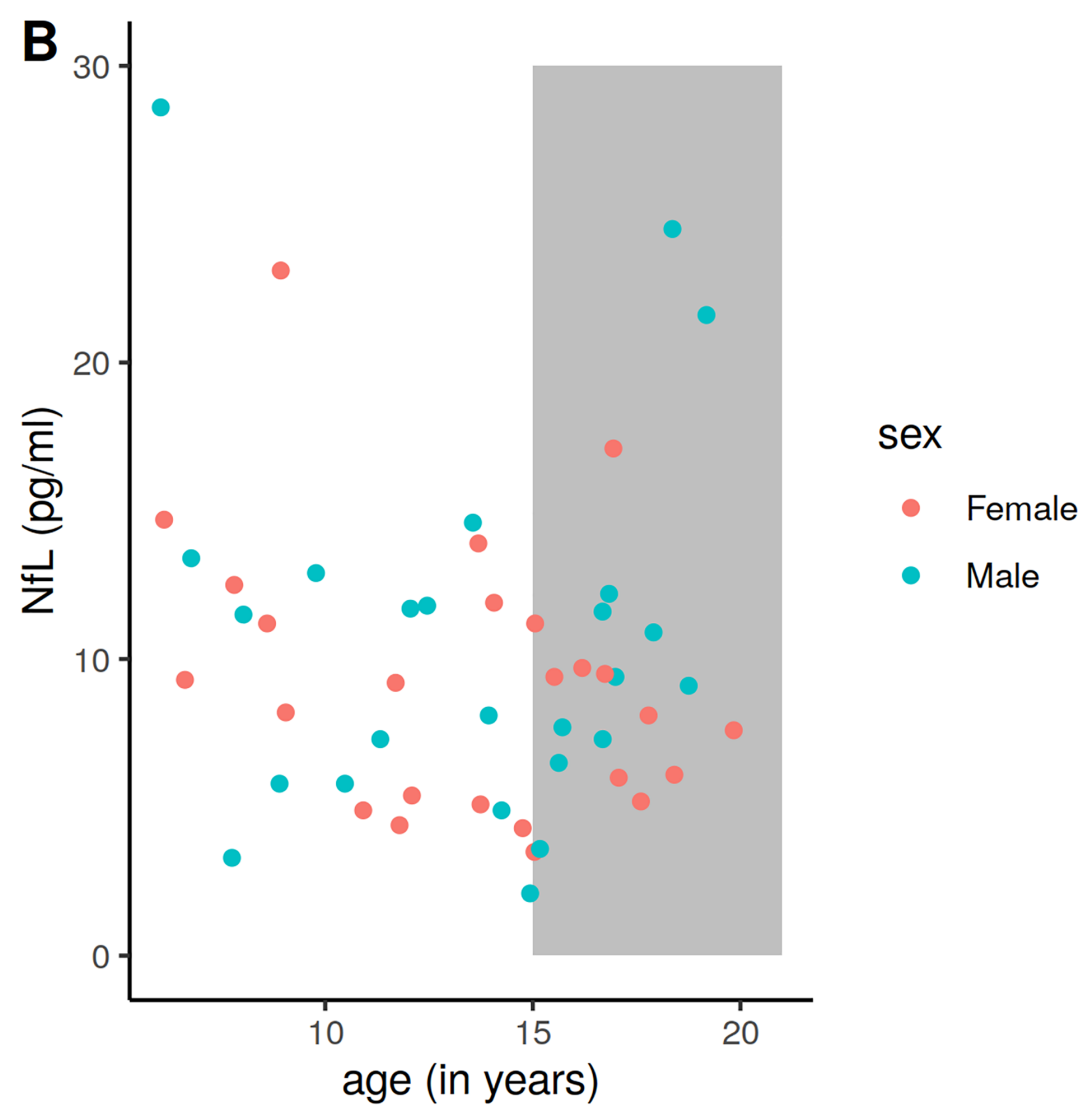

Supplement: Supplementary file 4 — Additional file 4. BDNF(A) and NfL (B) in HC plotted by age and gender. Data of patients aged >15 years are shaded. [file 13023_2020_1655_MOESM4_ESM.pdf]

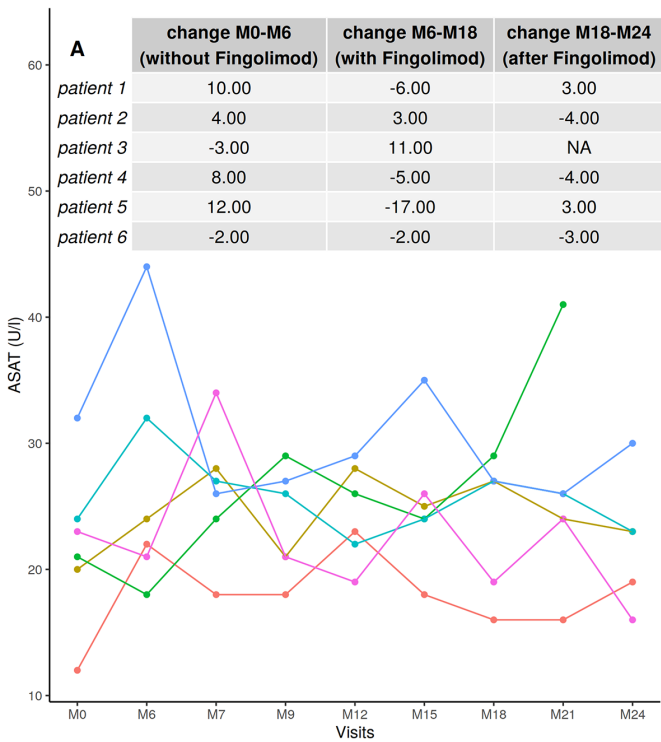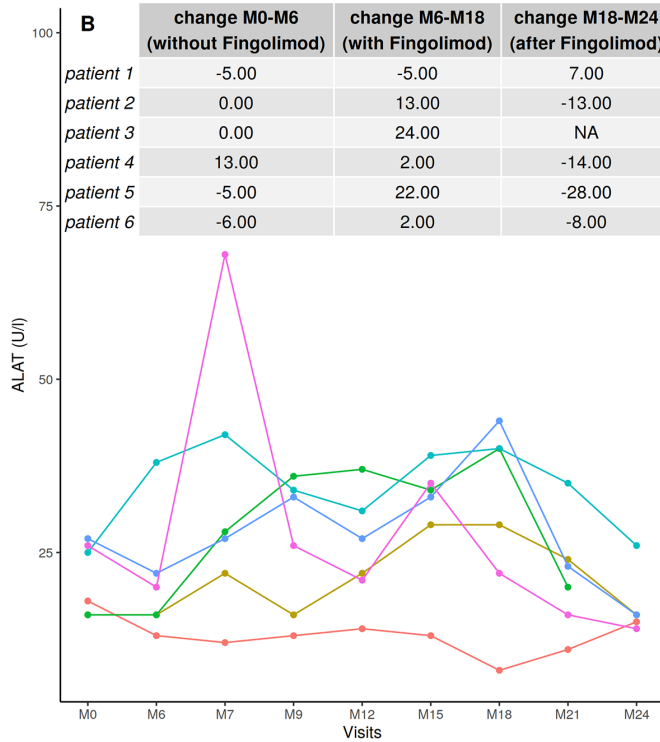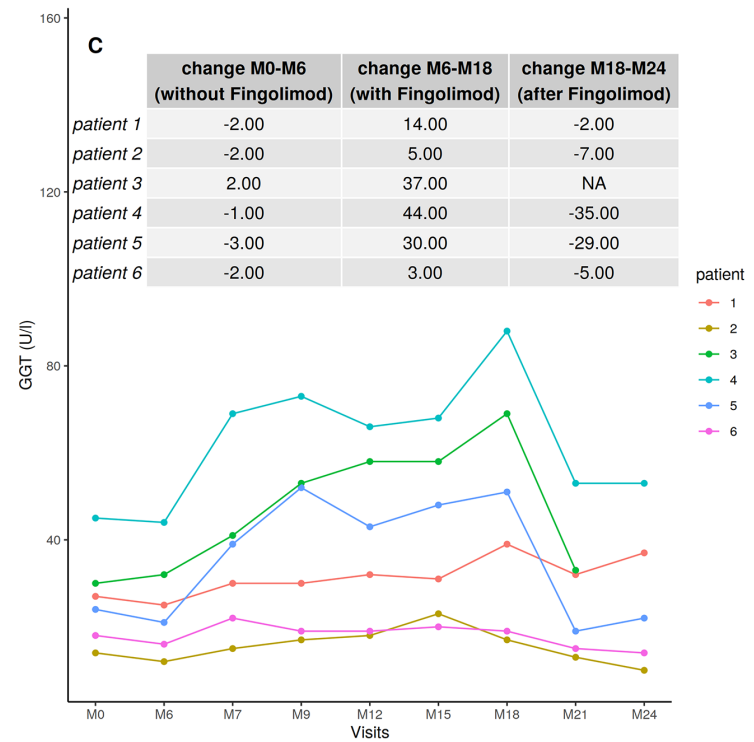

Supplement: Supplementary file 8 — Additional file 8. Liver enzymes over the study period. A: ASAT, B: ALAT, C: GGT. [file 13023_2020_1655_MOESM8_ESM.pdf]

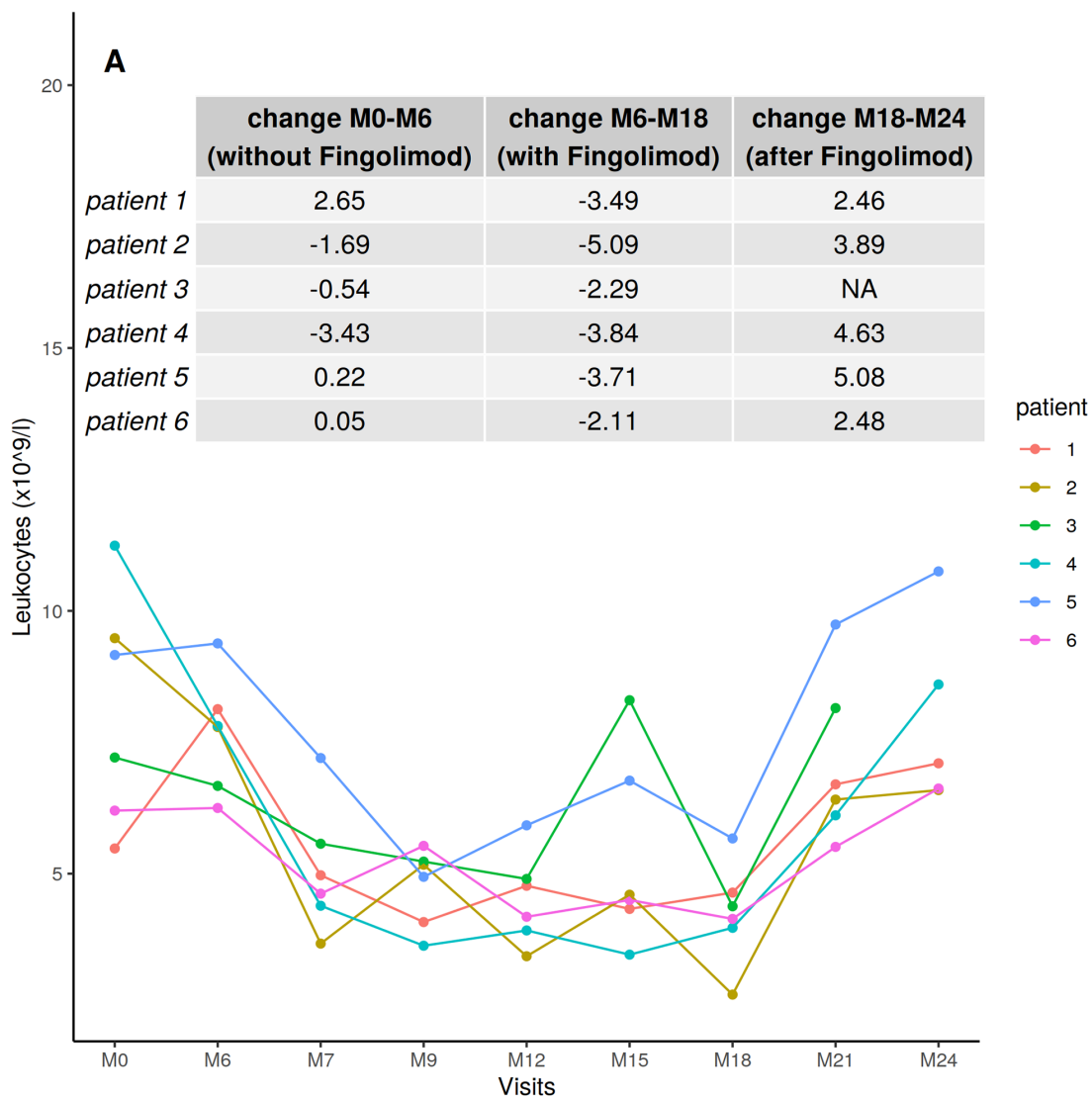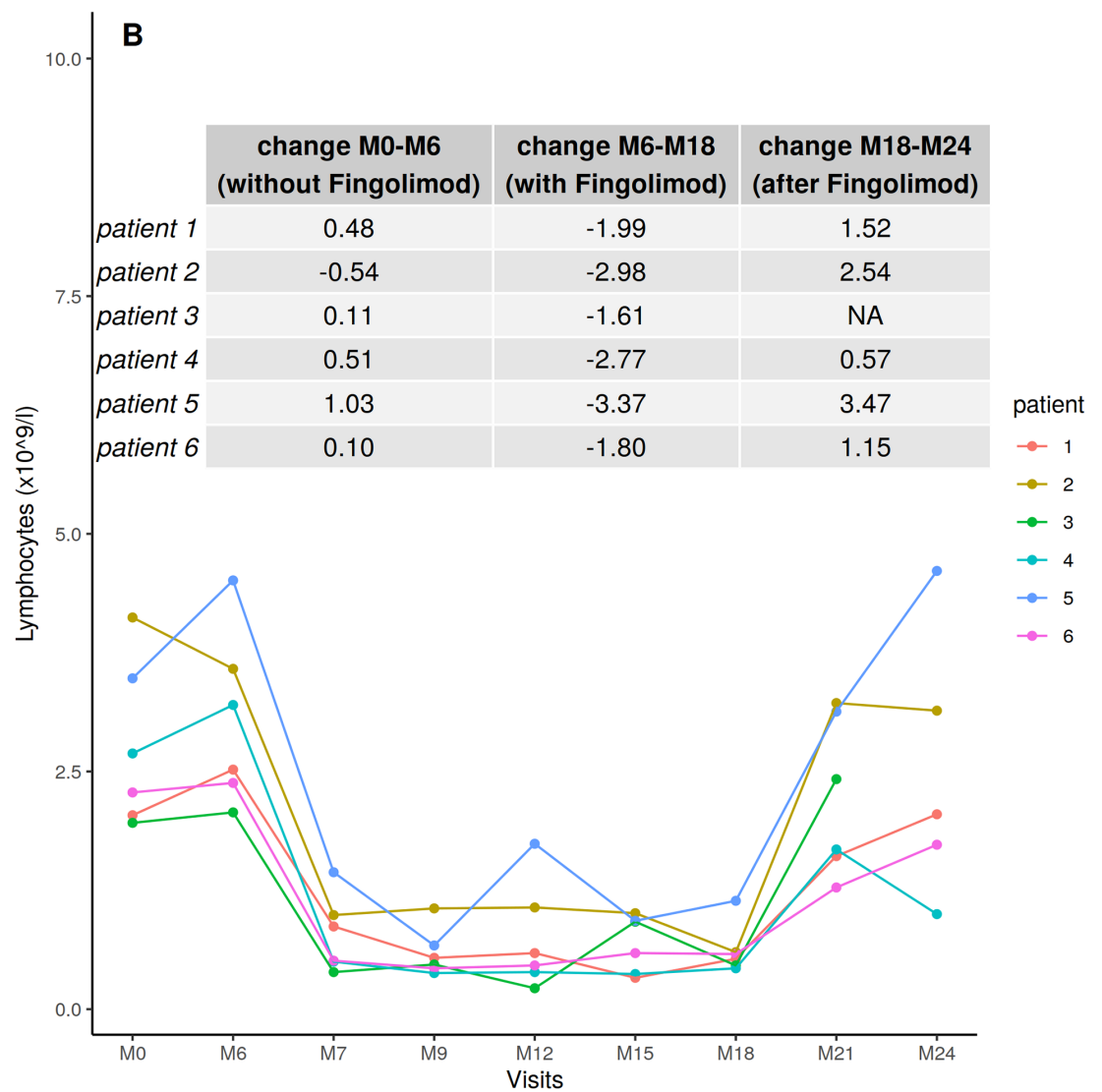

Supplement: Supplementary file 9 — Additional file 9. White blood cell count (leukocytes, A) and lymphocytes (B) over the study period. [file 13023_2020_1655_MOESM9_ESM.pdf]

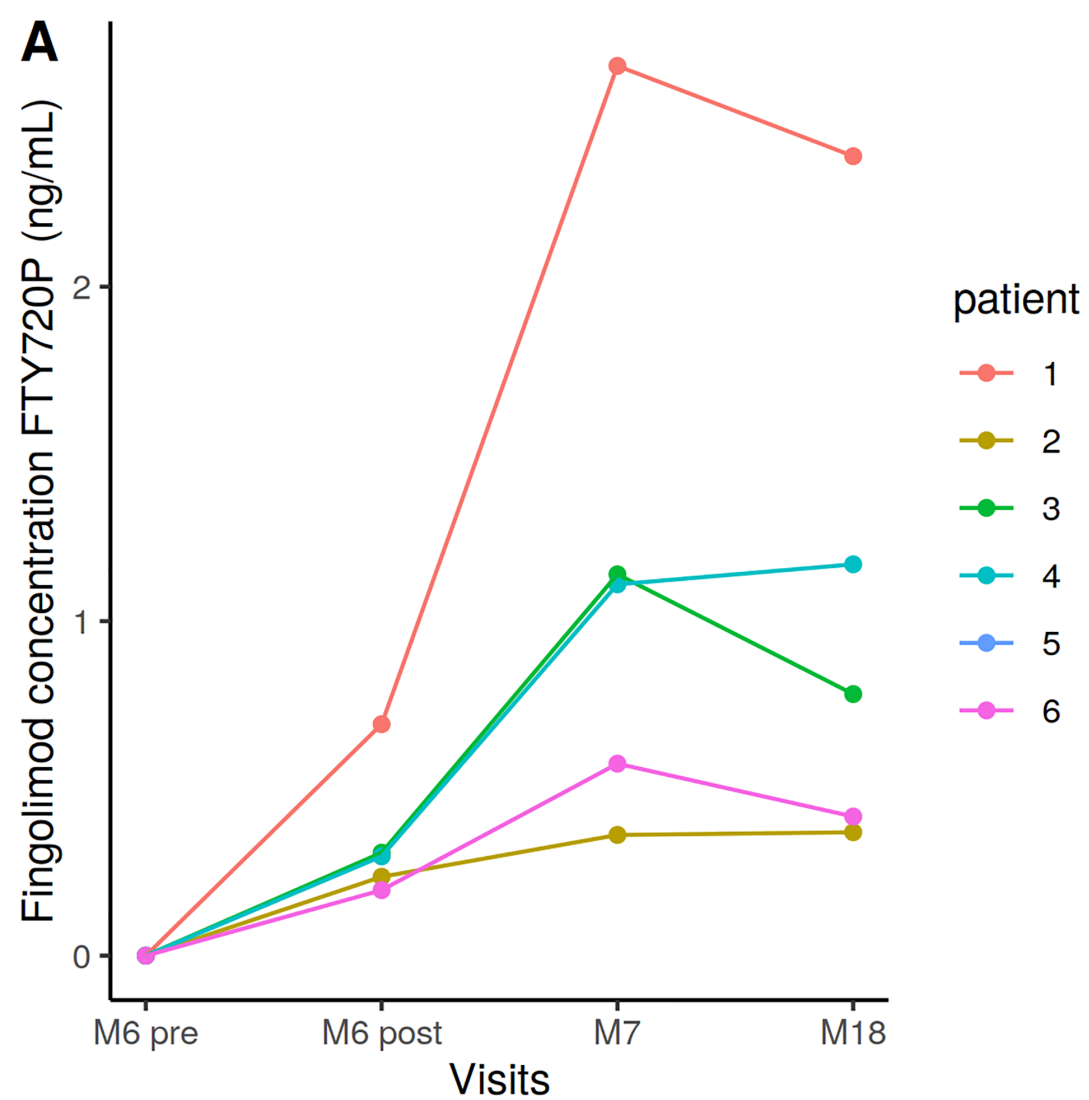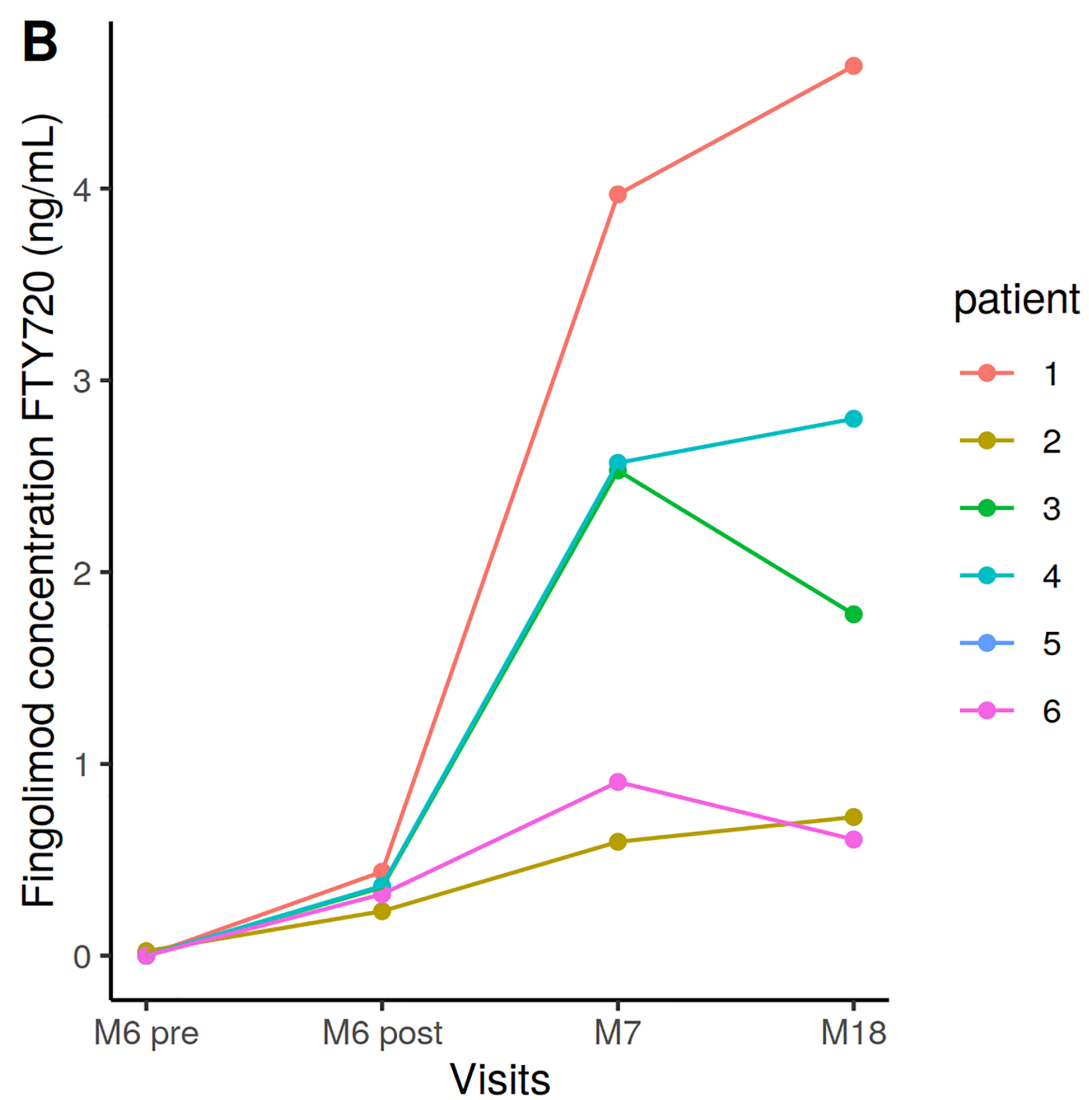

Supplement: Supplementary file 10 — Additional file 10. Concentration of FTY720P (A) and FTY720 (B) over the study period. [file 13023_2020_1655_MOESM10_ESM.pdf]
