# Supplementary material for: Fingolimod in children with Rett syndrome: the FINGORETT study
Source: Orphanet J Rare Dis. 2021 Jan 6;16:19. doi: 10.1186/s13023-020-01655-7 (PMC7789265; doi:10.1186/s13023-020-01655-7)
Supplement: Supplementary file 5 — Additional file 5. Change of BDNF and NfL before and under treatment. [file 13023_2020_1655_MOESM5_ESM.docx]

Additional file 5

|  | p-value | Change M0-M6 | Mean M0 | Mean  M6 | Mean M18 | Change M6-M18 | p-value |
| --- | --- | --- | --- | --- | --- | --- | --- |
| BDNF serum | 0.304 | -1.41, CI =  [-4.73;1.91] | 22.80 | 21.39 | 18.74 | -2.65, CI =  [-6.19;0.90] | 0.107 |
| BDNF CSF | 0.208 | -0.51, CI =  [-1.54;0.51] | 1.24 | 0.72 | 0.81 | 0.09, CI =  [-0.93;1.12] | 0.793 |
| NfL serum | 0.213 | -1.36, CI =  [-3.91;1.19] | 10.96 | 9.60 | 13.46 | 3.86, CI =  [-7.89;15.61] | 0.413 |
| NfL CSF | 0.782 | -24.00, CI =  [-276.23;228.23] | 462.20 | 438.20 | 430.38 | -7.83, CI =  [-71.88;56.23] | 0.723 |

*Additional file 5: Change of BDNF and NfL before and under treatment*
