# Supplementary material for: Fingolimod in children with Rett syndrome: the FINGORETT study
Source: Orphanet J Rare Dis. 2021 Jan 6;16:19. doi: 10.1186/s13023-020-01655-7 (PMC7789265; doi:10.1186/s13023-020-01655-7)
Supplement: Supplementary file 6 — Additional file 6. Changes of clinical scores over time. Change is indicated by absolute numbers of scores, eg. BL 7 and change over time -1, 3, -6: score changed from 7 to 6,9 and 3 respectively within the given time period. Changes over time in all scales and patients were not different in the intervals before during and after treatment. VABS = Vineland Adaptive Behaviour Scale; RSSS = Rett Severity Scale; HAS = Hand Apraxia Scale; M = Month of Study. [file 13023_2020_1655_MOESM6_ESM.docx]

Additional file 10

|  | **Individual Patients** | | | | | | |
| --- | --- | --- | --- | --- | --- | --- | --- |
| **Scales** | **Scores** | **001** | **002** | **003** | **004** | **005** | **006** |
| VABS  communication | BL-Score | 7 | 13 | 23 | 13 | 11 | 58 |
|  | Change M0-M6 (without Fingolimod) | 1 | 1 | -1 | 1 | -3 | -16 |
|  | Change M6-M18 (with Fingolimod) | 3 | 0 | -2 | 6 | 10 | 10 |
|  | Change M18-M24 (after Fingolimod) | 2 | -3 | -2 | 1 | 4 | 4 |
| VABS  Daily living | BL-Score | 11 | 9 | 14 | 12 | 8 | 40 |
|  | Change M0-M6 (without Fingolimod) | 2 | 1 | 9 | 1 | 1 | -2 |
|  | Change M6-M18 (with Fingolimod) | 0 | 2 | 2 | 3 | 1 | -4 |
|  | Change M18-M24 (after Fingolimod) | -5 | -3 | -3 | 5 | 1 | 0 |
| VABS  Socialization | BL-Score | 9 | 16 | 22 | 14 | 22 | 24 |
|  | Change M0-M6 (without Fingolimod) | 2 | 6 | 0 | 5 | -1 | 7 |
|  | Change M6-M18 (with Fingolimod) | 1 | -2 | 2 | 3 | 4 | -9 |
|  | Change M18-M24 (after Fingolimod) | 8 | -1 | -2 | 1 | -1 | 0 |
| VABS  Motor Functions | BL-Score | 14 | 6 | 30 | 8 | 4 | 38 |
|  | Change M0-M6 (without Fingolimod) | -1 | 6 | 0 | 2 | 0 | -15 |
|  | Change M6-M18 (with Fingolimod) | 9 | -2 | 2 | 6 | 2 | -1 |
|  | Change M18-M24 (after Fingolimod) | -2 | 0 | 0 | -5 | -4 | 3 |
| RSSS | BL-Score | 18 | 25 | 6 | 18 | 28 | 4 |
|  | Change M0-M6 (without Fingolimod) | -5 | 1 | 0 | -2 | -3 | -1 |
|  | Change M6-M18 (with Fingolimod) | 7 | 2 | 2 | -5 | 1 | -1 |
|  | Change M18-M24 (after Fingolimod) | -5 | -3 | -2 | 3 | -2 | 2 |
| HAS | BL-Score | 7 | 10 | 9 | 10 | 10 | 3 |
|  | Change M0-M6 (without Fingolimod) | -1 | -1 | -7 | -1 | 0 | 0 |
|  | Change M6-M18 (with Fingolimod) | 3 | 1 | -2 | -3 | -4 | 2 |
|  | Change M18-M24 (after Fingolimod) | -6 | 0 | 1 | 3 | 2 | -4 |

*Additional file 10:*  Change of clinical Scores over time. Change is indicated by absolute numbers of scores, eg. BL 7 and change over time -1, 3, -6: score changed from 7 to 6,9 and 3 respectively within the given time period. Changes over time in all scales were not different in the intervals before during and after treatment. VABS: Vineland Adaptive Behaviour Scale; RSSS: Rett Severity Scale; HAS: Hand Apraxia Scale; M: Month of Study
