# Supplementary material for: Fingolimod in children with Rett syndrome: the FINGORETT study
Source: Orphanet J Rare Dis. 2021 Jan 6;16:19. doi: 10.1186/s13023-020-01655-7 (PMC7789265; doi:10.1186/s13023-020-01655-7)
Supplement: Supplementary file 7 — Additional file 7. Brain volume change before and under treatment. [file 13023_2020_1655_MOESM7_ESM.docx]

Additional file 6

| Region | p-value | Change BL-M6 | Mean BL | Mean M6 | Mean M18 | Change M6-M18 | p-value |
| --- | --- | --- | --- | --- | --- | --- | --- |
| Thalamus | 0.711 | -73.17, CI = [-552.91;406.57] | 12987.67 | 12914.50 | 13074.83 | 160.33, CI = [-108.82;429.48 | 0.186 |
| Caudate | 0.66 | -47.83, CI = [-310.69;215.02] | 6683.33 | 6635.50 | 6651.67 | 16.17, CI = [-122.20;154.54] | 0.776 |
| Putamen | 0.782 | 49.00, CI = [-383.27;481.27] | 7549.67 | 7598.67 | 7600.50 | 1.83, CI = [-235.05;238.72] | 0.985 |
| Pallidum | 0.671 | 34.17, CI = [-160.55;228.88] | 2772.83 | 2807.00 | 2753.17 | -53.83, CI = [-158.27;50.60] | 0.242 |
| Hippocampus | 0.15 | 135.17, CI = [-68.99;339.32] | 5629.83 | 5765.00 | 5754.67 | -10.33, CI = [-230.52;209.85] | 0.909 |
| Amygdala | 0.789 | -28.00, CI = [-283.14;227.14] | 1811.83 | 1783.83 | 1960.33 | 176.50, CI = [57.91;295.09] | 0.012 |
| Accumbens | 0.995 | -0.17, CI = [-62.37;62.04] | 730.17 | 730.00 | 739.17 | 9.17, CI = [-34.03;52.36] | 0.609 |

Additional file 6: Brain volume change before and under treatment
